# Impact of beta-blocker usage on delirium in patients with sepsis in ICU: a cross-sectional study

**DOI:** 10.3389/fmed.2024.1458417

**Published:** 2024-09-13

**Authors:** Honglian Ouyang, Xiaoqi Wang, Dingwei Deng, Qianqian Wang, Yi Yu

**Affiliations:** ^1^Department of Critical Care Medicine, The Second Affiliated Hospital of Guangzhou University of Chinese Medicine, Guangzhou, Guangdong, China; ^2^Medical Intensive Care Unit, Sichuan Provincial Maternity and Child Health Care Hospital, Chengdu, Sichuan, China; ^3^The Affiliated Women's and Children's Hospital of Chengdu Medical College, Chengdu, Sichuan, China; ^4^Department of Pulmonary and Critical Care Medicine, The First Affiliated Hospital, Sun Yat-sen University, Guangzhou, Guangdong, China

**Keywords:** beta-blockers, delirium, sepsis, acute kidney injury, ICU stay, MIMIC-IV

## Abstract

**Introduction:**

Delirium in patients with sepsis can be life-threatening. This study aims to investigate the impact of the use of beta-blockers on the occurrence of delirium in patients with sepsis in the ICU by utilizing a comprehensive dataset.

**Methods:**

This is a cross-sectional study conducted using the data obtained from a single ICU in the USA. Patients diagnosed with sepsis and receiving beta-blockers were compared with those not receiving beta-blockers. Propensity score matching (PSM) and multiple regression analysis were employed to adjust for potential confounders.

**Results:**

Among the 19,660 patients hospitalized for sepsis, the beta-blocker and non-user groups comprised 13,119 (66.73%) and 6,541 (33.27%) patients, respectively. Multivariable logistic regression models revealed a significant reduction of 60% in 7-day delirium for beta-blocker users (OR = 0.40, 95% CI: 0.37–0.43, *p* < 0.001), for 30-day delirium (OR = 0.32, 95% CI: 0.29–0.35, *p* < 0.001), and for 90-day delirium (OR = 0.33, 95% CI: 0.30–0.35, *p* < 0.001). The PSM results further strengthen the validity of these findings. An analysis of safety issues demonstrated that beta-blockers may have an impact on the risk of acute kidney injury. However, following PSM, the results are not considered robust. Furthermore, there was no discernible change in the odds of renal replacement therapy and the length of ICU stays.

**Discussion:**

Our findings suggest a potential protective effect of beta-blockers against delirium in patients with sepsis. Nevertheless, the observational design limits causal inference, necessitating future randomized controlled trials to validate these findings.

## 1 Introduction

Delirium represents a prevalent form of organ dysfunction in critically ill adults and may substantially increase both morbidity and mortality rates. Some recent studies have indicated that more than half of all patients admitted to modern intensive care units (ICUs) will experience delirium at some point during their hospital stay (1–4). This is a critical issue owing to the independent association of delirium with an increase in mortality risk (5–7). Furthermore, the duration of delirium has been identified as the primary risk factor for subsequent cognitive impairment in individuals recovering from critical illness, often presenting as ICU-acquired delirium—a condition associated with substantial debilitation (3, 8–10). The onset of delirium is associated with prolonged hospitalizations and increased health care costs. Despite extensive research, however, no pharmacological agent has yet been developed that can show some efficacy in either treating or preventing delirium. Notably, current guidelines of the Society of Critical Care Medicine (SCCM) recommend against the routine use of dexmedetomidine, statins, or ketamine for preventing delirium in critically ill adults. These recommendations highlight the ongoing challenge of effectively managing delirium within ICU settings, necessitating further investigation into alternative therapeutic approaches (11).

Although the mechanism underlying delirium remains elusive, it has been suggested that delirium likely encompasses multiple pathways that are disrupted during critical illness, resulting in the impairment of normal cognitive function (12, 13). While various factors contributing to the pathophysiology of delirium have been recognized, numerous other aspects remain unidentified (14). Sepsis, arising from a dysregulated immune response after infection, is a significant public health concern (15). Various studies have suggested sepsis as one of the most imperative and robust risk factors for delirium (14, 16). Given the close association of both sepsis and delirium with increased morbidity and mortality rates (17–19), it becomes critical to prioritize the prevention and management of delirium in patients with sepsis.

Beta-blockers have been shown to be safe and effective in reducing 28-day mortality and controlling the ventricular rate in patients with sepsis post-fluid resuscitation, without any major adverse effects on tissue perfusion (20). Numerous systematic reviews have reported on beta-blocker therapy during sepsis (21–23). As beta-blockers play a pivotal role in sepsis management, their potential protective effects in patients with sepsis experiencing delirium should be investigated. Therefore, we herein conduct a retrospective study utilizing the Medical Information Mart for Intensive Care (MIMIC-IV) dataset for the 2008–2019 period. This investigation explores the association between the administration of beta-blockers and the onset of delirium in patients with sepsis.

## 2 Methods

We included patients with sepsis, both with and without exposure to beta-blockers, from the MIMIC-IV (version 2.2) database, a longitudinal single-center database covering the period spanning from 2008 to 2019 (24). Yi Yu, one of the authors, obtained permission to access the database (certificate ID: 6477678). The manuscript was prepared in accordance with the Guidelines for Strengthening the Reporting of Observational Studies in Epidemiology (25).

### 2.1 Study population and data extraction

Patients aged ≥18 years with a diagnosis of sepsis were included in the study. For patients with multiple ICU admissions, only the initial admission was considered. Patients with ICU stays shorter than 24 h or lacking essential information were excluded. Who received beta-blocker after the onset of delirium were also excluded. Delirium diagnosis was based on the Confusion Assessment Method for the ICU (CAM-ICU) criteria (26). Data on patient demographics, vital signs, laboratory results, comorbidities, clinical severity scores, and other admission details were collected.

### 2.2 Beta-blocker usage

Beta-blocker usage, defined as the use of beta-blockers at any time point, was determined by the presence of beta-blockers in the “Prescriptions” section of the MIMIC-IV database. This section mentioned beta-blockers such as acebutolol, atenolol, bisoprolol, esmolol, metoprolol, nadolol, nebivolol, propranolol, and timolol. The term “Pre-ICU” denotes the administration of beta-blockers exclusively prior to ICU admission, while “Post-ICU” signifies the use of beta-blockers exclusively following ICU admission. “Post + Pre ICU” refers to the utilization of beta-blockers both prior to and following admission to the ICU. “No use” indicates the absence of beta-blockers utilization.

### 2.3 Covariates

The risk factors for delirium among the patients with sepsis were documented (27, 28). The covariates analyzed included age, sex, body mass index (BMI), respiratory rate, white blood cell (WBC) count, hemoglobin level, platelet count, and glucose level (based on the first result upon admission to the ICU, or the average of multiple measurements over 24 h). The study collected data on health indicators, such as the SOFA score, and comorbid conditions, including cardiovascular diseases, kidney diseases, liver diseases, malignancy, neurological diseases, and chronic pulmonary diseases. Demographic information regarding race and marital status was also extracted. Multicollinearity was assessed by calculating the variance inflation factor (VIF) among variables involved. Multicollinearity was considered present if the VIF was greater than 2.

### 2.4 Outcome

The primary outcome was the incidence of delirium on 7, 30, and 90 days. The secondary outcomes included the length of the ICU stay, acute kidney injury (AKI), and the requirement for renal replacement therapy (RRT).

### 2.5 Statistical analysis

Baseline characteristics of patients across different groups were analyzed. Categorical data were presented as frequencies (percentages), while continuous variables were expressed as mean ± standard deviation or median (interquartile range) as appropriate. Statistical analyses involved the analysis of variance or Wilcoxon rank-sum tests to evaluate differences in continuous variables between groups. Chi-squared or Fisher’s exact tests were utilized for comparing categorical variables among the study cohort. Furthermore, consistent formatting and citation styles were adhered to throughout the study.

Missing data, constituting approximately 5% of vital signs and laboratory parameters, were imputed using the median. Given the low rates of missing data (0.5–8%) for height and weight, no imputation method was applied. A multivariate logistic regression analysis was conducted to evaluate the specific association between the usage of beta-blockers and delirium. The adjusted logistic model incorporated various covariates across six models. Additional analyses, adjusting for pertinent covariates, encompassed subgroup and interaction analyses. Propensity score matching (PSM) was implemented via a 1:1 nearest neighbor matching algorithm with a caliper width of 0.1 to enhance methodological rigor. PSM was used to reduce possible confounding factors that could affect the results and infer the true effect of the beta-blocker usage. Furthermore, the covariates mentioned above were selected to generate the propensity score. For deriving mortality odds ratios (OR), a multivariate logistic regression model with a robust variance estimator was employed. Linear regression analyses were conducted to investigate the relationship between beta-blocker usage and the length of ICU stay, while logistic regression analyses were conducted to assess associations between AKI and the necessity for RRT.

All statistical analyses were performed using STATA software (version 17.0), R packages (The R Foundation)^1^, and Free Statistics Software version 1.8 (29). Multiple imputations were conducted to handle missing values in logistic regression and model development. Statistical significance was set at *p* < 0.05. Two-tailed tests were employed consistently throughout the study.

## 3 Results

### 3.1 Participants

**Figure 1:**
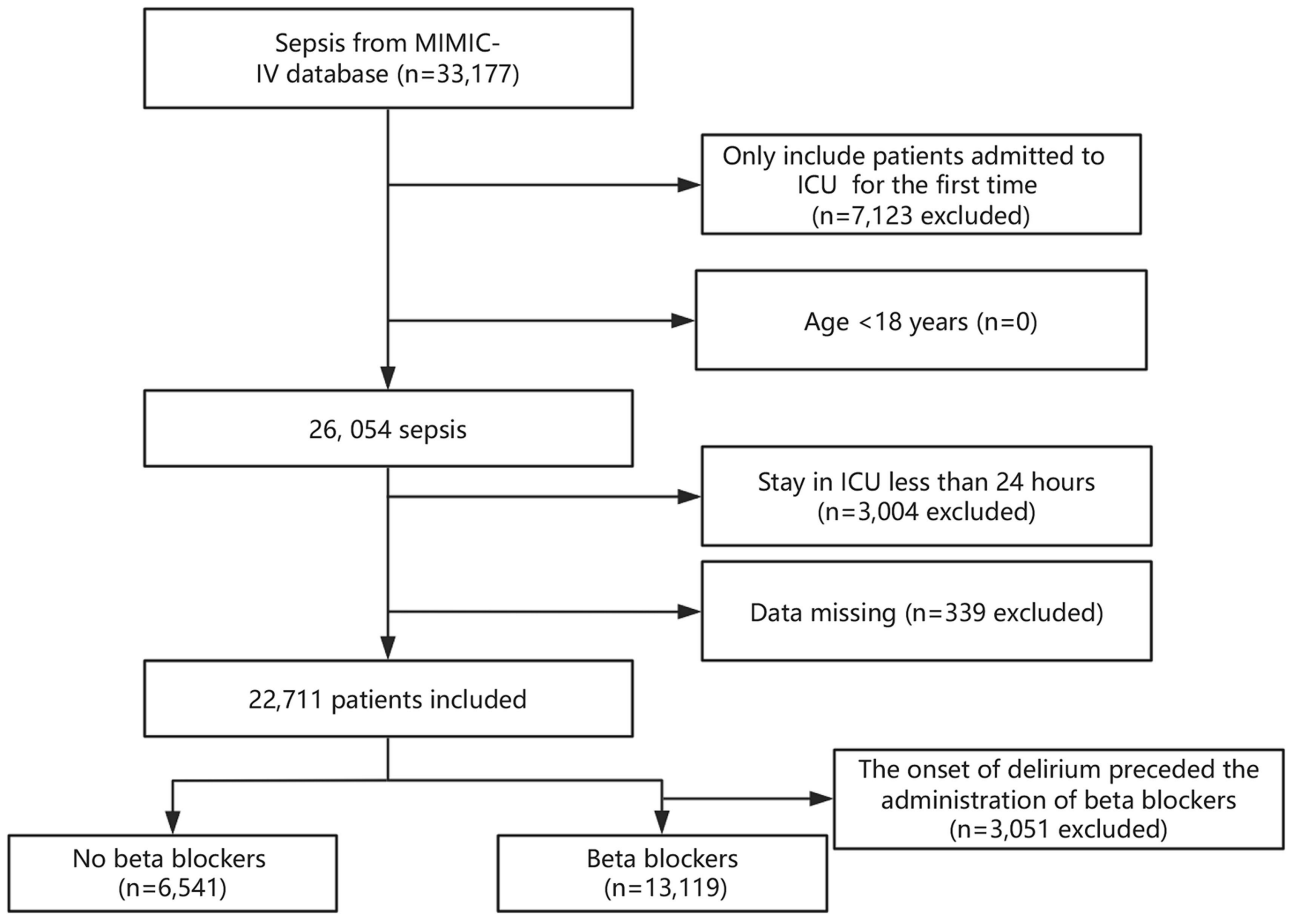
Flowchart of the selection of study participants.

A total of 33,177 patients met the criteria for sepsis. After excluding duplicate ICU admissions, individuals under 18 years of age, and those with an ICU stay of <24 h, the final study consisted of 19,660 patients. Figure 1 depicts the details of participant selection.

### 3.2 Baseline characteristics

The study included 19,660 patients, 58% of whom were men, with a mean age of 64.8 ± 16.3 years. Table 1 details the baseline characteristics of the patient included. Between-group comparisons revealed notable differences: the non-beta blocker group tended to be younger, had a higher proportion of women, a lower Charlson Comorbidity Index, and higher heart rates. The pre-ICU beta-blocker was found to be less effective than the non-beta-blocker alternative. This discrepancy may be attributed to the abrupt cessation of beta blocker administration (30, 31).

**Table 1:** Baseline characteristics of participants.

		Unmatched patients			Propensity-score–matched patients		
Variables	Total (*n* = 19,660)	Non beta blockers use (*n* = 6,541)	Beta blockers use (*n* = 13,119)	SMD	Non beta blockers use (*n* = 1,760)	Beta blockers use (*n* = 1,760)	SMD
Age, y	64.8 ± 16.3	58.8 ± 18.4	67.8 ± 14.2	0.549	65.5 ± 15.6	65.4 ± 14.5	<0.1
Sex, male, *n* (%)	11,404 (58.0)	3,486 (53.3)	7,918 (60.4)	0.014	944 (53.6)	980 (55.7)	<0.1
BMI, kg/m^2^	27.6 (24.5, 31.3)	27.6 (23.9, 30.1)	27.6 (24.8, 31.7)	0.027	27.6 (23.6, 31.2)	27.6 (24.0, 32.2)	<0.1
Race, *n* (%)				0.304			<0.1
White	13,488 (68.6)	4,012 (61.3)	9,476 (72.2)		1,127 (64)	1,118 (63.5)	
Black	1,636 (8.3)	548 (8.4)	1,088 (8.3)		179 (10.2)	185 (10.5)	
Others	4,536 (23.1)	1,981 (30.3)	2,555 (19.5)		454 (25.8)	457 (26)	
Marital status, *n* (%)				0.318			<0.1
Unmarried	10,680 (54.3)	4,141 (63.3)	6,539 (49.8)		1,131 (64.3)	1,109 (63)	
Married	8,980 (45.7)	2,400 (36.7)	6,580 (50.2)		629 (35.7)	669 (38.01)	
Insurance type, *n* (%)				0.176			<0.1
Medicaid	1,374 (7.0)	690 (10.5)	684 (5.2)		250 (7.1)	119 (6.8)	
Medicare	9,217 (46.9)	2,458 (37.6)	6,759 (51.5)		1,699 (48.3)	832 (47.3)	
other	9,069 (46.1)	3,393 (51.9)	5,676 (43.3)		1,571 (44.6)	809 (46)	
WBC (×10^9^)	13.1 ± 9.0	13.3 ± 10.3	13.0 ± 8.2	0.07	13.3 ± 8.3	13.3 ± 9.4	<0.1
Hb (g/L)	10.6 ± 2.0	10.8 ± 2.2	10.4 ± 1.9	0.259	10.4 ± 2.2	10.4 ± 2.1	<0.1
PLT (×10^9^)	204.6 ± 111.4	206.9 ± 112.6	203.4 ± 110.8	0.068	205.2 ± 112.2	201.1 ± 118.9	<0.1
Respiration rate (bpm)	19.6 ± 4.1	20.1 ± 4.4	19.3 ± 3.8	0.012	20.1 ± 4.1	20.1 ± 4.1	<0.1
Heart rate (bpm)	86.5 ± 16.0	87.9 ± 16.8	85.7 ± 15.5	0.007	87.6 ± 16.6	87.5 ± 16.5	<0.1
MAP, mean ± SD	76.7 ± 10.3	77.2 ± 10.7	76.4 ± 10.0	0.084	77.5 ± 11.0	77.4 ± 10.7	<0.1
Lactate (mmol/L)	2.4 ± 1.8	2.5 ± 2.0	2.4 ± 1.6	0.137	2.6 ± 2.2	2.6 ± 1.8	<0.1
BUN, median (IQR)	20.0 (14.0, 33.5)	18.5 (12.5, 31.5)	21.0 (14.5, 34.8)	0.263	22.0 (14.5, 38.0)	23.0 (15.0, 39.5)	<0.1
Cr, median (IQR)	1.0 (0.8, 1.5)	1.0 (0.7, 1.5)	1.0 (0.8, 1.6)	0.228	1.0 (0.7, 1.8)	1.0 (0.8, 1.7)	<0.1
Glucose (mg/dl)	128.5 (108.4, 159.0)	128.5 (107.5, 161.0)	128.5 (109.0, 158.0)	0.021	133.0 (111.0, 172.5)	134.0 (110.5, 166.5)	<0.1
Charlson comorbidity index	5.6 ± 2.9	4.8 ± 3.1	6.0 ± 2.7	0.552	5.9 ± 2.8	5.9 ± 2.6	<0.1
SOFA score	5.6 ± 3.3	5.8 ± 3.8	5.6 ± 3.1	0.022	6.3 ± 3.8	6.4 ± 3.7	<0.1
SAPS II	39.2 ± 14.4	38.0 ± 15.8	39.8 ± 13.6	0.238	42.6 ± 14.5	42.7 ± 14.1	<0.1
MI *n* (%)	2,940 (15.0)	441 (6.7)	2,499 (19)	0.336	192 (10.9)	202 (11.5)	<0.1
CHF, *n* (%)	5,183 (26.4)	850 (13)	4,333 (33)	0.602	350 (19.9)	400 (22.7)	<0.1
CBVD, *n* (%)	2,529 (12.9)	800 (12.2)	1,729 (13.2)	0.009	312 (17.7)	327 (18.6)	<0.1
Dementia, *n* (%)	728 (3.7)	337 (5.2)	391 (3)	0.062	123 (7)	130 (7.4)	<0.1
CPD, *n* (%)	4,834 (24.6)	1,490 (22.8)	3,344 (25.5)	0.071	458 (26)	459 (26.1)	<0.1
Severe liver disease, *n* (%)	1,075 (5.5)	490 (7.5)	585 (4.5)	0.135	166 (9.4)	170 (9.7)	<0.1
Diabetes, *n* (%)				0.356			<0.1
Non	1,3,813 (70.3)	5,186 (79.3)	8,627 (65.8)		1,227 (69.7)	1,228 (69.8)	
Without complications	4,145 (21.1)	982 (15)	3,163 (24.1)		353 (20.1)	344 (19.5)	
With complications	1,702 (8.7)	373 (5.7)	1,329 (10.1)		180 (10.2)	188 (10.7)	
Renal disease, *n* (%)	4,038 (20.5)	809 (12.4)	3,229 (24.6)	0.463	326 (18.5)	334 (19)	<0.1
Malignant cancer, *n* (%)	2,648 (13.5)	1,014 (15.5)	1,634 (12.5)	0.015	270 (15.3)	251 (14.3)	<0.1
Dexmedetomidine, *n* (%)	2,924 (14.9)	1,007 (15.4)	1,917 (14.6)	0.089	490 (27.8)	487 (27.7)	<0.1
Midazolam, *n* (%)	4,665 (23.7)	1,814 (27.7)	2,851 (21.7)	0.08	515 (29.3)	519 (29.5)	<0.1
ICU stay, days	2.8 (1.7, 5.4)	3.0 (1.8, 5.9)	2.7 (1.6, 5.2)	0.036	4.6 (2.5, 9.2)	4.5 (2.3, 8.8)	<0.1
AKI in 7 day, *n* (%)	14,217 (72.3)	4,293 (65.6)	9,924 (75.6)	0.202	1,383 (78.6)	1,380 (78.4)	<0.1
RRT, *n* (%)	1,086 (5.5)	396 (6.1)	690 (5.3)	0.093	161 (9.1)	165 (9.4)	<0.1
Delirium onset from ICU admission, day	1.8 (0.3, 6.5)	1.2 (0.2, 3.8)	2.5 (0.5, 12.3)	0.343	1.3 (0.2, 4.1)	2.0 (0.4, 6.9)	<0.1

For each variable, mean ± standard deviation, median (interquartile range), or number (percentage) is reported (as appropriate).

SMD, standardized mean difference; BMI, body mass index; WBC, white blood cells; HB, hemoglobin; PLT, platelets; MAP, mean arterial pressure; BUN, blood urea nitrogen; Scr, serum creatinine; SOFA, sequential organ failure assessment; SAPS II, simplified acute physiology score II; MI, myocardial infarct; CHF, congestive heart failure; CBVD, cerebrovascular disease; CPD, chronic pulmonary disease; ICU, intensive care unit; AKI, acute kidney injury; RRT, renal replacement therapy.

### 3.3 Relationship between beta-blocker usage and delirium

In the univariate analysis, the beta-blocker usage was significantly associated with lower delirium rates compared to those observed with non-beta blocker usage, with ORs of 0.47 (95% CI, 0.44–0.50, *p* < 0.001) for 7-day delirium, 0.39 (95% CI, 0.36–0.42, *p* < 0.001) for 30-day delirium, and 0.40 (95% CI, 0.37–0.43, *p* < 0.001) for 90-day delirium (Table 2). Subsequently, in the extended multivariate logistic regression analysis (Table 2), the beta-blocker usage consistently demonstrated significant ORs across all models (ranging from 0.32 to 0.47, *p* < 0.001 for all). Following adjustment for all covariates listed in Table 2, beta-blocker users showed a 60% reduction in the risk of 7-day delirium (OR = 0.40, 95% CI: 0.37–0.43, *p* < 0.001, model 6). Similarly, a 68% lower risk was observed for 30-day delirium (OR = 0.32, 95% CI: 0.29–0.35, *p* < 0.001, model 6) and a 67% lower risk was observed for 90-day delirium rates among beta-blocker users (OR = 0.33, 95% CI: 0.30–0.35, *p* < 0.001, model 6). These results highlight the robustness of the analytical models employed.

**Table 2:** Beta blockers usage for delirium in ICU patients with sepsis.

	OR	95% CI	*p* value
7 day			
Model 1	0.47	0.44–0.50	< 0.001
Model 2	0.45	0.42–0.48	< 0.001
Model 3	0.46	0.43–0.49	< 0.001
Model 4	0.46	0.43–0.49	< 0.001
Model 5	0.46	0.42–0.49	< 0.001
Model 6	0.40	0.37–0.43	<0.001
PSM	0.50	0.47–0.54	<0.001
30 day			
Model 1	0.39	0.36–0.42	<0.001
Model 2	0.36	0.34–0.39	<0.001
Model 3	0.38	0.35–0.41	<0.001
Model 4	0.38	0.35–0.40	<0.001
Model 5	0.38	0.35–0.41	<0.001
Model 6	0.32	0.29–0.35	<0.001
PSM	0.54	0.42–0.70	<0.001
90 day			
Model 1	0.40	0.37–0.43	<0.001
Model 2	0.37	0.35–0.40	<0.001
Model 3	0.39	0.36–0.42	<0.001
Model 4	0.39	0.36–0.41	<0.001
Model 5	0.39	0.36–0.42	<0.001
Model 6	0.33	0.30–0.35	<0.001
PSM	0.59	0.44–0.80	<0.001

OR, odds ratio; CI, confidence interval; PSM, propensity score matching.

Model 1: Not adjusted.

Model 2: Model 1 adjusted for age, sex, BMI.

Model 3: Model 2 adjusted for insurance, marital status, race.

Model 4: Model 3 adjusted for heart rate, MAP, respiration rate, lactate, WBC, HB, PLT, BUN, Cr.

Model 5: Model 4 adjusted for myocardial infarct, congestive heart failure, cerebrovascular disease, dementia, chronic pulmonary disease, diabetes, renal disease, malignant cancer, severe liver disease, SOFA score.

Model 6: Model 5 adjusted for dexmedetomidine, midazolam, propofol, AKI on 7th day, RRT, ICU stay, SAPS II, 90-day mortality.

PSM: adjusted for Model 6.

### 3.4 Relationship between beta-blocker usage and other outcomes

After controlling for all covariates from Tables 3, 4, the beta-blocker usage was not significantly related to the length of ICU stay (*β* = −2.27, 95% CI: −6.31 to 1.77). Our findings did not suggest that prolonged ICU stays can be attributed to beta-blockers (*β* = −2.24, 95% CI: −12.27 to 7.79) following PSM. Stratification of beta-blocker administration based on timing indicated that its initiation post-ICU admission was associated with a prolonged ICU duration, whereas pre-ICU initiation was linked to a reduction in ICU stays (Table 3).

**Table 3:** Beta blockers usage and ICU stay (hours).

	Model 1	Model 2	PSM
Variable	β (95%CI)		
No beta blockers	0 (Ref)	0 (Ref)	0 (Ref)
Beta blockers	−5.12 (−9.27 to −0.96)	−2.27 (−6.31 to 1.77)	−2.24 (−12.27 to 7.79)
Time of administration			
No use	0 (Ref)	0 (Ref)	0 (Ref)
Pre- ICU	−1.95 (−8.77 to 4.88)	−5.13 (−11.39 to 1.13)	−24.21 (−38.42 to −10)
Post-ICU	4.64 (−0.39 to 9.67)	4.41 (−0.32 to 9.14)	40.57 (24.93 to 56.22)
Pre-ICU and post-ICU	−16.07 (−21.09 to −11.06)	−8.8 (−13.66 to −3.94)	−12.51 (−25.79 to 0.77)

Ref, reference; CI, confidence interval; PSM, propensity score matching.

Model 1: Not adjusted.

Model 2: Adjusted for age, sex, BMI, insurance, marital status, race, heart rate, MAP, respiration rate, Lactate, WBC, HB, PLT, myocardial infarct, congestive heart failure, cerebrovascular disease, dementia, chronic pulmonary disease, diabetes mellitus, renal disease, malignant cancer, severe liver disease, SOFA score, dexmedetomidine, midazolam, propofol, AKI on 7^th^ day, RRT, BUN, Cr, SAPS II, 90-day mortality.

**Table 4:** Beta blockers usage and other outcomes.

	AKI			RRT		
	Model 1	Model 2	PSM	Model 1	Model 2	PSM
Variable	OR (95%CI)					
No beta blockers	1 (Ref)					
Beta blockers	1.63 (1.52–1.74)	1.37 (1.26–1.48)	0.94 (0.78–1.12)	0.86 (0.76–0.98)	1.1 (0.92–1.31)	1.12 (0.83–1.51)
Time of administration						
No use	1 (Ref)					
Pre-ICU	1.97 (1.75–2.21)	1.30 (1.14–1.48)	0.85 (0.65–1.10)	1.83 (1.54–2.17)	1.16 (0.92–1.46)	1.02 (0.68–1.53)
Post-ICU	1.54 (1.42–1.67)	1.37 (1.25–1.51)	1.07 (0.80–1.44)	0.67 (0.57–0.79)	1.02 (0.82–1.28)	1.43 (0.9–2.28)
Pre-ICU and post-ICU	1.6 (1.47–1.73)	1.38 (1.26–1.52)	0.93 (0.74–1.18)	0.7 (0.59–0.83)	1.04 (0.96–1.11)	1.04 (0.67–1.61)

OR, odds ratio; Ref, Reference; CI, Confidence interval; PSM, Propensity score matching.

Model 1: Not adjusted.

Model 2: Adjusted for age, sex, BMI, insurance, marital status, race, heart rate, MAP, respiration rate, Lactate, WBC, HB, PLT, myocardial infarct, congestive heart failure, cerebrovascular disease, dementia, chronic pulmonary disease, diabetes mellitus, renal disease, malignant cancer, severe liver disease, SOFA score, dexmedetomidine, midazolam, propofol, BUN, Cr, ICU stay, SAPS II, 90-day mortality.

PSM: adjusted for Model 2.

Beta-blocker usage was associated with a 37% increase in the risk of AKI on the 7th day (OR = 1.37, 95% CI: 1.26–1.48). However, our findings did not suggest that beta-blocker usage elevates the risk of AKI (OR = 0.94, 95% CI: 0.78–1.12) after PSM. Additionally, beta-blocker usage did not correlate with an increased likelihood of requiring RRT (OR = 1.1, 95% CI: 0.92–1.31). Stratifying beta-blocker administration by timing indicated that beta-blocker usage was not associated with an increased likelihood of requiring RRT. The implementation of PSM reinforced the reliability of these findings (Table 4).

### 3.5 Subgroup and sensitivity analyses

**Figure 2:**
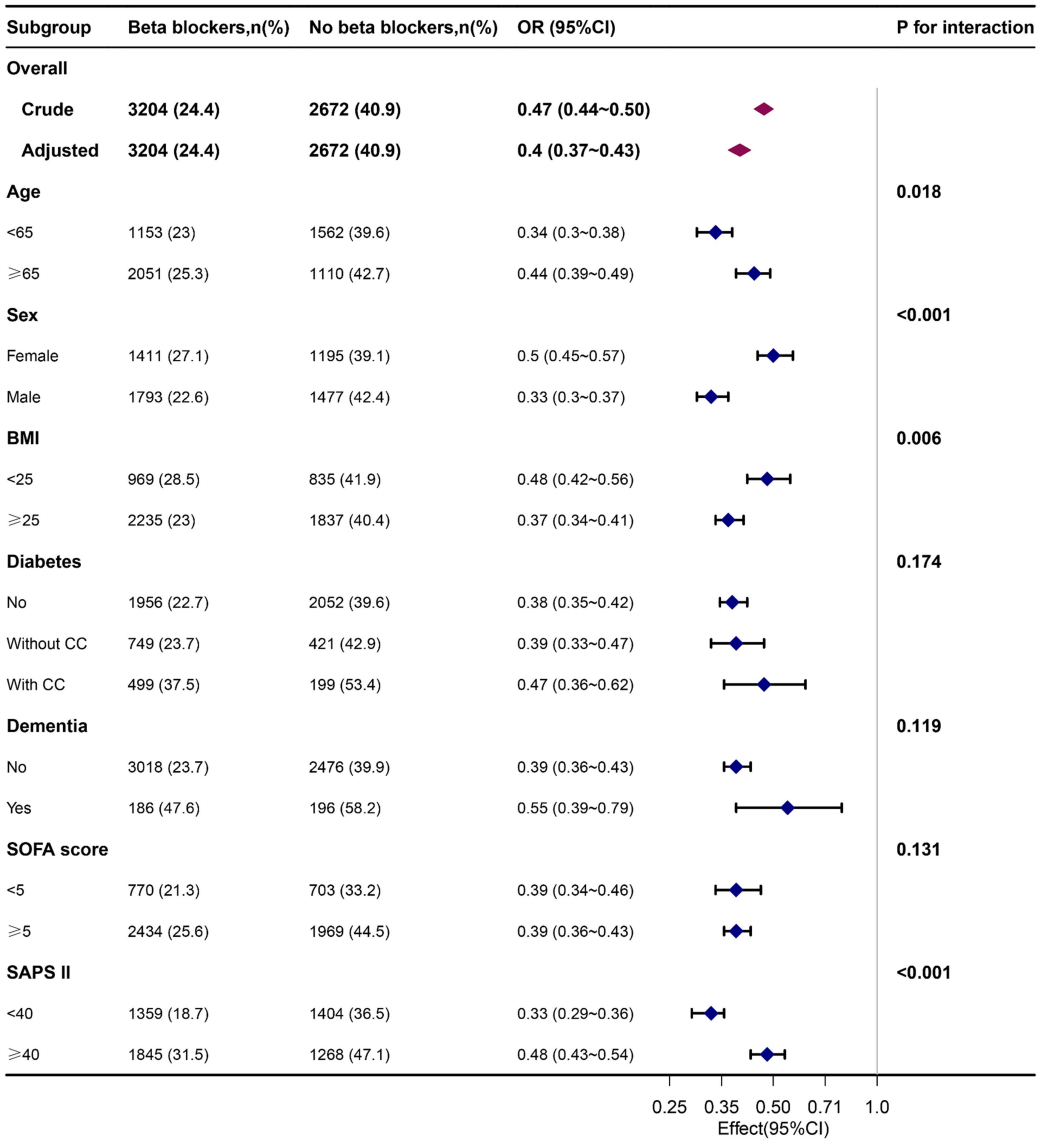
Associations of delirium in patients who received beta-blockers with those who did not receive them on the 7th day, by baseline characteristics. Each stratification was adjusted for all factors, excluding the stratified factor itself. OR, odd ratio; BMI, body mass index; CC, complication; SOFA, Sequential organ failure assessment.

Subgroup analysis confirmed the robust and consistent nature of our findings. Specifically, beta-blocker usage showed a more pronounced protective effect in individuals younger than 65 years, men, BMI ≥ 25 kg/m^2^ and SAPS II < 40. However, no other significant interactions were observed in subgroup analyses for 7-day delirium (*P* for interaction >0.05) (Figure 2). A similar trend was obtained with 30-day and 90-day delirium outcomes (Supplementary Figures S1, S2).

Following PSM, the study consisted of 1,760 well-matched pairs, with no significant differences in key indicators between the two matched groups. The robustness of our findings is affirmed across logistic regression models. Specifically, among the users of beta-blockers, those initiating treatment post-ICU exhibited a lower OR (0.28, 95% CI, 0.25–0.31), whereas pre-ICU initiators showed a higher OR (1.1, 95% CI, 0.99–1.22). Patients using beta-blockers both pre- and post-ICU demonstrated a lower OR (0.55, 95% CI, 0.50–0.60), with all trends statistically significant (*p* < 0.001) for 7-day delirium. Similar patterns were observed for 30-day and 90-day delirium outcomes (Supplementary Table S1). A subgroup analysis of specific beta-blocker types revealed that metoprolol and atenolol consistently reduced delirium at 7, 30, and 90 days, findings that were robust across PSM analyses. However, other categories of beta-blockers did not consistently demonstrate significant effects across different time points and models (Supplementary Table S2). There are no significant differences in mortality between the groups after PSM (Supplementary Table S3).

## 4 Discussion

### 4.1 Main findings

Our study is the most comprehensive investigation to date on the impact of beta-blockers on delirium in patients with sepsis. We found that patients with sepsis who were administered beta-blockers had a lower adjusted prevalence of delirium at 7, 30, and 90 days compared to those who did not receive beta-blockers. Importantly, these results remained consistent even after adjusting for potential confounders using PSM. We did not observe any increase in the risk of AKI or need for RRT associated with beta-blocker usage, nor did we find a significant association with the length of the ICU stay. Our findings suggest a potential protective effect of beta-blockers against delirium in patients with sepsis.

### 4.2 Effects of beta-blocker usage on delirium in patients with sepsis

It has been suggested that beta-blockers mitigate the sepsis-induced hypermetabolic state, cardiac dysfunction, and coagulopathy (32). Our findings indicate that patients with sepsis administered beta-blockers had a decreased adjusted incidence of delirium at 7, 30, and 90 days compared to those who did not receive beta-blockers.

These findings are in contrast to those reported in previous studies. A retrospective cohort study of 490 patients, all aged ≥70 years, admitted for hip fracture revealed no link between beta-blocker administration and the onset of postoperative delirium (33). Similarly, a study of 2,648 patients who received beta-blockers within 24 h before cardiac surgery, controlled for potential confounders, did not identify an independent association between beta-blocker usage and postoperative delirium (34). In contrast, another study reported that the preoperative administration of beta-blockers increased the odds of postoperative delirium by 2.06 times among patients who underwent vascular surgery (35). Our study findings diverge from those of previous research, potentially owing to variations in the study population. Specifically, our study targeted patients with sepsis, in whom the effects of beta-blockers might differ from those in other patient cohorts. We theorize that beta-blockers may act by attenuating the inflammatory cascade response mediated by catecholamine surge and enhancing cytokine release (36–38). Previous studies have demonstrated that the administration of beta-blockers reduces the release of proinflammatory factors, such as interleukin-6 and tumor necrosis factor-α (39, 40). However, the previous studies did not address the temporal dimension of delirium or employ logistic regression analyses, unlike our study, which integrated both methodologies, thereby offering more holistic insights related to delirium. In addition, our study further categorized the administration of beta-blockers based on timing and classification.

Pre-ICU admission beta-blocker usage was associated with an increased risk of delirium, whereas post-ICU and combined pre- and post-ICU usage were linked to a decreased risk of delirium. These findings are consistent with the 2014 ACC/AHA Guidelines on Perioperative Cardiovascular Evaluation and Management of Patients Undergoing Noncardiac Surgery, which advocate for the continuation of beta-blocker therapy in patients under regular preoperative treatment and its initiation in intermediate-to-high-risk patients (41).

### 4.3 Effects of beta-blocker usage on other outcomes for patients with sepsis

The use of beta-blockers significantly increased the risk of AKI by the 7th day (OR = 1.37, 95% CI: 1.26–1.48). However, no association was found with an increased use of RRT (OR = 1.1, 95% CI: 0.92–1.31). Importantly, our findings did not indicate that beta-blocker administration results in heightened AKI rates, increased RRT usage, or prolonged ICU stays following PSM.

Notably, the cardio-inhibitory effects of beta-blockers may compromise the cardiac compensatory reserve, which is essential for renal perfusion (42), thereby potentially precipitating renal failure. A study involving 2,972 adult recipients of living donor liver transplantation (LDLT) from January 2012 to July 2022 concluded, following PSM analyses, that there was no significant difference in the incidence of AKI between groups. Preoperative beta-blocker use did not correlate with AKI following LDLT (43). Although the impact of beta-blockers on AKI has been studied across diverse patient cohorts (44–46), the research remains limited on their influence on AKI incidence in patients with sepsis. In septic shock, esmolol-induced beta-blockade significantly enhances the pressure dependency of renal blood flow compared to renal perfusion pressure, potentially impairing renal autoregulation. Discontinuation of esmolol suggests the potential for the reversibility of this effect (47). Therefore, additional investigation is warranted to ascertain the potential contribution of beta-blockers to AKI and the need for RRT in patients with sepsis.

### 4.4 Strengths of our study

Our study has several major strengths. Firstly, we utilized a comprehensive and publicly accessible database, ensuring the reliability and comprehensiveness of our data. Secondly, to our knowledge, no prior research has specifically investigated the impact of beta-blocker usage on delirium risk in patients with sepsis. Our findings provide compelling and definitive evidence that beta-blocker administration significantly reduces the occurrence of delirium in this population. Thirdly, we employed multiple regression analyses and PSM to establish the robustness and credibility of our study outcomes. This rigorous analytical approach enhances the internal validity and credibility of our findings. Lastly, given the widespread use of beta-blockers for cardiovascular conditions, our results have implications and applicability beyond the septic patient population specifically.

### 4.5 Limitations of our study

This study represents the most comprehensive investigation to date on the use of beta-blockers in ICU patients with sepsis. However, it is subject to several limitations. A major constraint is the notable variability in beta-blocker dosages and treatment durations, potentially introducing heterogeneity into the reported data. Secondly, caution is advised in generalizing the implications of our study, as it was conducted using data from a single ICU facility in the USA. Thirdly, certain factors that could contribute to sepsis-related delirium were not accounted for in the available studies, such as the adequacy of antibiotic therapy, volume resuscitation, history of alcohol consumption, and phosphorus levels, making further analysis on these aspects unfeasible. Fourthly, due to the observational nature of this study, it did not employ the optimal methodology for assessing drug effects. As this study employed a cross-sectional design, the temporal interval between the administration of beta-blockers and the onset of delirium may have varied. Consequently, the statistical analyses may have been susceptible to the bias inherent in observational studies. Future randomized controlled trials should employ a more suitable approach. Despite these limitations, the large sample size and the use of PSM partially mitigate these constraints.

## 5 Conclusion

This analysis clearly demonstrates that administering beta-blockers decreases the occurrence of delirium in patients with sepsis. However, as this evidence mainly derives from non-randomized studies, there may be limitations in interpreting and applying these findings more broadly. Therefore, further research is necessary to thoroughly understand this relationship.

## Data Availability

Publicly available datasets were analyzed in this study. This data can be found here: all data in the article can be obtained from MlMIC-IV database (https://mimic.physionet.org/).

## Footnotes

^1^ http://www.R-project.org

## Supplementary material

SUPPLEMENTARY FIGURE S1 Associations of delirium in patients who received beta-blockers with those who did not receive them on the 30th day, by baseline characteristics. Each stratification was adjusted for all factors excluding the stratified factor itself.

SUPPLEMENTARY FIGURE S2 Associations of delirium in patients who received beta-blockers with those who did not receive them on the 90th day by baseline characteristics. Each stratification was adjusted for all factors excluding the stratified factor itself.
