# Supplementary material for: Impact of beta-blocker usage on delirium in patients with sepsis in ICU: a cross-sectional study
Source: Front Med (Lausanne). 2024 Sep 13;11:1458417. doi: 10.3389/fmed.2024.1458417 (PMC11427366; doi:10.3389/fmed.2024.1458417)
Supplement: SUPPLEMENTARY FIGURE S1 — Associations of delirium in patients who received beta-blockers with those who did not receive them on the 30th day, by baseline characteristics. Each stratification was adjusted for all factors excluding the stratified factor itself. [file Table_1.docx]

**Table 1S.** Classification of beta blockers according to their time of administration for delirium in ICU patients with sepsis.

|  | **Non-use** | **Pre-ICU** | **Post-ICU** | **Pre- and post-ICU** | **P for trend** |
| --- | --- | --- | --- | --- | --- |
|  | | **OR (95% CI)** | | |  |
| **7 Day** | | | | | |
| **Model 1** | 1(Ref) | 1.22 (1.14-1.31) | 0.3 (0.28-0.33) | 0.52 (0.49-0.56) | <0.001 |
| **Model 2** | 1(Ref) | 1.18 (1.1-1.27) | 0.29 (0.27-0.32) | 0.5 (0.47-0.54) | <0.001 |
| **Model 3** | 1(Ref) | 1.21 (1.12-1.3) | 0.3 (0.28-0.33) | 0.52 (0.49-0.56) | <0.001 |
| **Model 4** | 1(Ref) | 1.14 (1.05-1.22) | 0.31 (0.29-0.33) | 0.52 (0.49-0.56) | <0.001 |
| **Model 5** | 1(Ref) | 1.1 (1.02-1.19) | 0.31 (0.29-0.34) | 0.54 (0.5-0.58) | <0.001 |
| **Model 6** | 1(Ref) | 1.07 (0.99-1.16) | 0.28 (0.26-0.3) | 0.49 (0.46-0.53) | <0.001 |
| **PSM** | 1(Ref) | 1.1 (0.99-1.22) | 0.28 (0.25-0.31) | 0.55 (0.50-0.60) | <0.001 |
| **30 Day** | | | | | |
| **Model 1** | 1(Ref) | 1.19 (1.11-1.29) | 0.24 (0.22-0.26) | 0.43 (0.4-0.46) | <0.001 |
| **Model 2** | 1(Ref) | 1.14 (1.05-1.23) | 0.23 (0.21-0.25) | 0.4 (0.37-0.43) | <0.001 |
| **Model 3** | 1(Ref) | 1.19 (1.1-1.28) | 0.23 (0.21-0.26) | 0.42 (0.39-0.45) | <0.001 |
| **Model 4** | 1(Ref) | 1.14 (1.05-1.23) | 0.24 (0.22-0.26) | 0.42 (0.39-0.46) | <0.001 |
| **Model 5** | 1(Ref) | 1.11 (1.02-1.2) | 0.25 (0.23-0.27) | 0.45 (0.41-0.48) | <0.001 |
| **Model 6** | 1(Ref) | 1.08 (1-1.17) | 0.22 (0.2-0.24) | 0.41 (0.38-0.44) | <0.001 |
| **PSM** | 1(Ref) | 1.1 (0.99-1.23) | 0.23 (0.21-0.27) | 0.46 (0.42-0.51) | <0.001 |
| **90 Day** | | | | | |
| **Model 1** | 1(Ref) | 1.2 (1.11-1.29) | 0.25 (0.23-0.27) | 0.44 (0.41-0.47) | <0.001 |
| **Model 2** | 1(Ref) | 1.14 (1.06-1.23) | 0.24 (0.22-0.26) | 0.41 (0.39-0.45) | <0.001 |
| **Model 3** | 1(Ref) | 1.19 (1.1-1.28) | 0.24 (0.22-0.26) | 0.43 (0.4-0.47) | <0.001 |
| **Model 4** | 1(Ref) | 1.14 (1.05-1.23) | 0.25 (0.23-0.27) | 0.44 (0.41-0.47) | <0.001 |
| **Model 5** | 1(Ref) | 1.11 (1.02-1.2) | 0.26 (0.23-0.28) | 0.46 (0.43-0.5) | <0.001 |
| **Model 6** | 1(Ref) | 1.08 (0.99-1.17) | 0.23 (0.21-0.25) | 0.42 (0.39-0.45) | <0.001 |
| **PSM** | 1(Ref) | 1.11 (1-1.23) | 0.24 (0.21-0.27) | 0.47 (0.43-0.52) | <0.001 |

Abbreviations: Pre-ICU, Pre-ICU admission, Post-ICU, Post-ICU admission; OR, odds ratio; CI, confidence interval; PSM, propensity score matching.

**Model 1:** Not adjusted.

**Model 2: Model 1** adjusted for age, sex, BMI.

**Model 3: Model 2** adjusted for insurance, marital status, race.

**Model 4**: **Model 3** adjusted for heart rate, MAP, respiration rate, lactate, WBC, HB, PLT, BUN, Cr.

**Model 5**: **Model 4** adjusted for myocardial infarct, congestive heart failure, cerebrovascular disease, dementia, chronic pulmonary disease, diabetes mellitus, renal disease, malignant cancer, severe liver disease, SOFA score.

**Model 6**: **Model 5** adjusted for dexmedetomidine, midazolam, propofol, AKI on 7th day, RRT, ICU stay, SAPS II, 90-day mortality.

**PSM**: adjusted for Model 6.
