# Supplementary material for: Impact of beta-blocker usage on delirium in patients with sepsis in ICU: a cross-sectional study
Source: Front Med (Lausanne). 2024 Sep 13;11:1458417. doi: 10.3389/fmed.2024.1458417 (PMC11427366; doi:10.3389/fmed.2024.1458417)
Supplement: SUPPLEMENTARY FIGURE S2 — Associations of delirium in patients who received beta-blockers with those who did not receive them on the 90th day by baseline characteristics. Each stratification was adjusted for all factors excluding the stratified factor itself. [file Table_2.docx]

**Table 2S.** Subclassification of beta blockers for delirium in ICU patients with sepsis.

|  | **7 day** | | |  | **30 day** | | |  | **90 day** | | |
| --- | --- | --- | --- | --- | --- | --- | --- | --- | --- | --- | --- |
|  | **Model 1** | **Model 2** | **PSM** |  | **Model 1** | **Model 2** | **PSM** |  | **Model 1** | **Model 2** | **PSM** |
|  | **OR (95%CI)** | | | | | | | | | | |
| **None** | 1(Ref) | | | | | | | | | | |
| **Metoprolol** | 0.48 (0.46-0.51) | 0.44 (0.41-0.47) | 0.46 (0.43-0.49) |  | 0.41 (0.39-0.44) | 0.38 (0.36-0.41) | 0.41 (0.38-0.44) |  | 0.42 (0.4-0.45) | 0.39 (0.37-0.42) | 0.42 (0.38-0.45) |
| **Esmolol** | 0.98 (0.77-1.26) | 0.68 (0.53-0.88) | 0.64 (0.44-0.92) |  | 0.87 (0.66-1.14) | 0.61 (0.46-0.79) | 0.59 (0.4-0.88) |  | 0.89 (0.68-1.16) | 0.61 (0.47-0.8) | 0.62 (0.43-0.91) |
| **Atenolol** | 0.57 (0.49-0.67) | 0.6 (0.52-0.7) | 0.67 (0.54-0.82) |  | 0.53 (0.45-0.62) | 0.56 (0.48-0.66) | 0.64 (0.51-0.8) |  | 0.52 (0.44-0.61) | 0.55 (0.47-0.65) | 0.62 (0.5-0.78) |
| **Propranolol** | 0.99 (0.8-1.24) | 0.82 (0.65-1.02) | 0.83 (0.62-1.11) |  | 0.89 (0.7-1.13) | 0.75 (0.59-0.95) | 0.78 (0.58-1.06) |  | 0.9 (0.71-1.14) | 0.75 (0.59-0.95) | 0.8 (0.6-1.08) |
| **Timolol** | 0.85 (0.7-1.03) | 0.78 (0.64-0.95) | 0.84 (0.62-1.13) |  | 0.74 (0.6-0.92) | 0.68 (0.55-0.84) | 0.78 (0.56-1.07) |  | 0.78 (0.63-0.96) | 0.72 (0.58-0.89) | 0.83 (0.61-1.13) |
| **Others** | 0.66 (0.57-0.76) | 0.59 (0.51-0.68) | 0.68 (0.56-0.82) |  | 0.57 (0.49-0.67) | 0.52 (0.44-0.61) | 0.59 (0.48-0.73) |  | 0.57 (0.49-0.66) | 0.52 (0.44-0.61) | 0.59 (0.48-0.73) |

Abbreviations: OR, odds ratio; Ref, reference; CI, confidence interval; PSM, propensity score matching.

**Model 1:** Not adjusted.

**Model 2:** Adjusted for age, sex, BMI, insurance, marital status, race, heart rate, MAP, respiration rate, lactate, WBC, HB, PLT, myocardial infarct, congestive heart failure, cerebrovascular disease, dementia, chronic pulmonary disease, diabetes mellitus, renal disease, malignant cancer, severe liver disease, SOFA score, dexmedetomidine, midazolam, propofol, AKI on 7th day, RRT, BUN, Cr, ICU stay, SAPS II, 90-day mortality.

**PSM**: adjusted for Model 2.
