# Supplementary material for: Impact of beta-blocker usage on delirium in patients with sepsis in ICU: a cross-sectional study
Source: Front Med (Lausanne). 2024 Sep 13;11:1458417. doi: 10.3389/fmed.2024.1458417 (PMC11427366; doi:10.3389/fmed.2024.1458417)
Supplement: Supplementary file 3 [file Table_3.docx]

**Table 3S:** Mortality rate of participants.

|  | | **Unmatched Patients** | | |  | **Propensity-Score–Matched Patients** | | |
| --- | --- | --- | --- | --- | --- | --- | --- | --- |
| **Variables** | **Total**  **(n = 19,660)** | **Non beta blockers use**  **(n = 6,541)** | **Beta blockers use**  **(n = 13,119)** | **P** |  | **Non beta blockers use**  **(n =1,760)** | **Beta blockers use**  **(n =1,760)** | **P** |
| 30-day mortality, n (%) | 3,923 (20.0) | 1,539 (23.5) | 2,384 (18.2) | < 0.001 |  | 495 (28.1) | 503 (28.6) | 0.765 |
| 90-day mortality, n (%) | 4,213 (21.4) | 1,615 (24.7) | 2,598 (19.8) | < 0.001 |  | 535 (30.4) | 560 (31.8) | 0.363 |
